# States, law, and the regulation of controversial health-related claims: consolidating a research agenda between disciplines and contexts

**DOI:** 10.12688/wellcomeopenres.24597.1

**Published:** 2025-08-06

**Authors:** Emilie Cloatre, Martyn Pickersgill, Caesar A. Atuire, Mairead Enright, Phoebe Friesen, Patricia Kingori, `Tidiane Ndoye, Nayeli Urquiza-Haas

**Affiliations:** 1 Law, King's College London The Dickson Poon School of Law, London, England, UK; 2 The University of Edinburgh Usher Institute of Population Health Sciences and Informatics, Edinburgh, Scotland, UK; 3 University of Oxford Centre for Tropical Medicine and Global Health, Oxford, England, UK; 4 Department of Philosophy, University of Ghana, Accra, Greater Accra Region, Ghana; 5 School of Law, Loughborough University, Loughborough, England, UK; 6 Department of Equity, Ethics and Policy, McGill University, Montreal, Québec, Canada; 7 University of Oxford Ethox Centre, Oxford, England, UK; 8 Department of Sociology, Universite Cheikh Anta Diop de Dakar, Dakar, Dakar Region, Senegal; 9 Lancaster University Law School, Lancaster, England, UK

**Keywords:** Misinformation; health-claims; law and regulation

## Abstract

Stories of unproven, disproven, or misleading health-related claims, and their impact on individual and public health, are commonplace around the world. Disquiet about such claims is ubiquitous and growing within public, clinical, scientific, and policy discourse, with law commonly presented as having an important role to play in addressing concerns. Action, though, requires regulators to account for competing considerations, including fundamental freedoms, cultural diversity, and the potential for law to exacerbate inequalities. The latter is particularly significant when assessing the veracity of marginalised beliefs. In practice, legal decision-makers walk a fine line between everyday tolerance and occasional intervention. Yet, legal research pertinent to these issues is surprisingly limited. Here, we argue that new knowledge, methods, and collaborations are needed to better understand how regulatory interventions relevant to contested claims are constituted; how they operate in practice; and how they relate to different political and social processes - including acts of public resistance (like campaigns and protests). Only once we are collectively equipped with such critical knowledge of the current nature and possibilities of regulatory relations will it be possible to collectively design more imaginative and inclusive legal responses.

## Introduction

Stories of unproven, disproven, or misleading health-related claims, and their impact on individual and public health, are commonplace around the world. These intersect wide-ranging areas, from mental health (e.g. unlicensed practitioners working with paying clients to deliver their own bespoke 'therapies') and reproductive health (e.g., advertising of so-called ‘abortion reversal’), and from the everyday (e.g., dieting advice) to the life-threatening (e.g., bleach for 'treating' COVID). They encompass disparate actors from social media influencers to religious lobbies ( Baker & Rojek, 2020; Peters, 2007). Disquiet about controversial health claims is ubiquitous and growing within public, clinical, scientific, and policy discourse, with law commonly presented as having an important role to play in addressing concerns ( Lavorgna & Horsburgh, 2020).

Sometimes, though not always, debates around unproven, disproven or misleading claims are framed as being about misinformation or indeed disinformation (with the distinction between these being that disinformation is assumed to be intentionally misleading; Purnat & Clark, 2025). However, although both terms have been widely embraced and have evident value for focusing minds and attention, we consider that a less dichotomous, or a more expansive, framing is also necessary to consider. This is for three key reasons. First, the terms misinformation and disinformation suggest clear distinctions between truth and falsity, that are not always easy to draw in health contexts ( Kingori, 2021). In these, the un/proven is negotiated over time, on occasion to be later contested or revisited, particularly in less researched domains. Even though some claims might easily be dismissed as demonstrably false or misleading, other disruptive claims might be better characterised as contested or debatable. Indeed, what should count as evidence in health to ascertain truth is often at the core of intense debates ( Friesen, 2019). Second, not all unproven - or even misleading or disproven - claims are seen as equally problematic by policymakers. Instead, enunciations regarded as misinformation/disinformation are a smaller category of such claims, constituted in part through the perceived level of state engagement they require. Third, while claims deemed unproven may be designed to deceive, including for personal or financial profit, others are more akin to forms of resistance from groups that are actively neglected by biomedicine. The sociopolitical configurations from which such claims emerge, then, and the ways in which they feed or respond to different types of social inequalities, are relevant considerations for strategies of regulation and governance. Accordingly, more fluid categorisations than misinformation and disinformation may be needed to understand different dynamics shaping the emergence, circulating, and instantiation of controversial health-related claims, and the role of the state within these.

Regardless of framing, since the Covid-19 ‘infodemic’ ( Caulfield, 2020; Cosentino, 2023) regulators are often asked to address claims deemed problematic more effectively, including through the use of hard law. Action, though, requires regulators to account for competing considerations, including fundamental freedoms, cultural diversity, and the potential for law to exacerbate inequalities. This is a particularly significant consideration when assessing the veracity of marginalised beliefs ( Johnson, 2005). In practice, legal decision-makers walk a fine line between everyday tolerance and occasional intervention.

Legal research pertinent to these issues is surprisingly limited. Some scholars have aptly demonstrated the complexity of relevant legal regimes ( Katsirea, 2018; Röttgers-Witz & De Boer, 2021) and the urgency of adjusting regulatory responses ( Kadakia *et al.*, 2023; Mamak, 2021). Yet, the focus of much scholarship to-date has predominantly been on specific cases within jurisdictions in the Global North, and have been largely doctrinal or prescriptive. More empirically-led, critical approaches on the other hand are rarer. Meanwhile, pertinent social science research on health misinformation has paid limited attention to the role of law and regulation in synergistically and reciprocally shaping the social processes through which unproven, disproven or misleading claims arise and circulate ( Kirkland, 2023).

We argue that new knowledge, methods, and collaborations are needed to better understand how regulatory interventions relevant to such claims are constituted; how they operate in practice; and how they relate to different political and social processes - including acts of public resistance (like campaigns and protests). Only once we are collectively equipped with such critical knowledge of the current nature and possibilities of regulatory relations will it be possible to collectively design more imaginative and inclusive legal responses.

## A fragmented legal picture

State decisions about when and how to intervene against unproven, disproven or misleading health-related claims involve multiple considerations. Some claims can be harmful, including those related to life-threatening conditions (e.g., disproven cancer ‘cures’), or those aiming to ‘treat’ something that is not an illness (e.g., so-called ‘conversion therapy’). In these cases, regulation is paramount to protect those whose circumstances place them in a situation of vulnerability ( Freckelton, 2020). Nevertheless, what regulation looks like - or *could* or *should* look like - varies.

Indeed, regulation can come at a cost. In particular, it can encroach on fundamental freedoms, disrupt legitimate dissent, or silence cultural beliefs - sometimes disproportionately ( Vese, 2022). Even where claims seem antithetic to conventional biomedical epistemologies, they may serve distinct purposes; for example, nourishing different networks of care, or helping those for whom ‘evidence-based medicines’ are unavailable or ineffective ( Bivins, 2021; Brosnan *et al.*, 2023; Friesen & Blease, 2018). Finally, regulation can have unexpected effects. It can, for instance, drive claims and the practices they enact ‘underground’, and thus increase the risks for users ( Klein & Potter, 2018), or it may overlook negative impacts on disadvantaged groups (e.g., Indigenous health networks ( Marks & Cohen, 2021). In so doing, patterns of inequality can be reinforced while remaining hidden or dismissed from the gaze of lawmakers ( Crosby, 2019; Paton, 2009).

State responses to particular claims can take a variety of forms. Sometimes, the boundaries of (un)acceptable health-related claims are drawn in highly visible ways, through legislative change or during moments of crisis ( Charruau, 2020), bringing the above tensions to the fore. More commonly, they are shaped through technical, ad hoc decisions, including via courts or regulatory agencies ( Rizzi *et al.*, 2021; Rogers, 2007). In that respect, ‘regulation’ in this field can be inconsistent, and the logics of different sub-domains and actors can be difficult to reconcile.

It is also notable that the definition and boundaries of what regulation or law look like, and indeed their empirical recognition, are further complicated by the participation of private actors in state decision-making. This can be in direct and traceable ways, such as when state actors participate in delegated or informal modes of regulation. The role of social media platforms is an obvious example, but such alternative regulatory actors may take more surprising forms; for instance, when the Catholic Church certifies healing miracles in secular France ( Szabo, 2002). Less directly, transnational actors - including commercial entities – influence, the boundaries of state action through reports, recommendations, and lobbying ( De La Brosse *et al.*, 2022). Nationally and globally, both corporate and community campaigners lobby for legal reform or trigger legal action, sometimes challenging the claims of health professionals or government officials ( Cano-Orón & Lopera-Pareja, 2021; Wepukhulu, 2022).

Within this fragmented landscape, the diverse rationales behind regulatory decisions are difficult to trace. Yet, they are also key to understanding the social meaning of different interventions, the assumptions that underpin them, and the various impacts they are likely to have. Regulatory approaches – at national, supranational, or local institutional levels - are shaped through different notions of social, health, and epistemic norms and different ideas of social (in)justice. These require systematic attention in order to understand how contrasting approaches are co-constituted. Accordingly, direct comparisons and critical assessments of the regulation of unproven, disproven or misleading health-related claims are difficult without in-depth and cross-cutting interdisciplinary research of the kind that so far has not been substantively undertaken.

## The ebb and flow of state tolerance

Rather than a fully predictable and rational system of rules, we argue that law and regulation in this field are best approached as the drawing of fast-changing and sometimes surprising lines of tolerance. States handle unproven, disproven, and misleading health-related claims as a balancing act, drawing dotted lines between what can be tolerated, and in what context, and what should be deterred (and when and how). However, different states balance tolerance and intervention differently, undergirded by contrasting legal, socio-cultural, and political environments, even when addressing shared concerns (e.g., vaccine-related misinformation). Moreover, this balancing is not always explicit, but rather unfolds through everyday political life.

Biomedically unproven claims - including those grounded in popular wisdom, faith, or magic - often coexist with official public health advice, with limited disruption from state authorities. Of course, tolerance is not unbounded; yet, its limits and justifications are challenging to map. Even strategies explicitly designed to tackle ‘health misinformation’ tend to be selective. Some unproven claims, perhaps because they are seen as benign, do not tend to be visible within policy discourses centred on misinformation. In other cases, it is more surprising not to see some highly contested claims being apprehended as part of these discourses ( Tan, 2021; Wahlberg, 2007). Often, definitions of what claims are legally acceptable or not, and why, are unclear ( McClean & Moore, 2014). For instance, when are faith-healing claims deemed bona fide or fraudulent? Is advice on TikTok benign lifestyle content or harmful pseudo-medical misinformation? When do recommendations for dieting become seen as so harmfully restrictive that they need countering?

There is great diversity in how different states challenge, tolerate, or accommodate unproven, disproven, or misleading health-related claims. Even what different states, or different regulators within states, come to define as ‘misinformation’, or when this misinformation warrants state intervention, seems to vary significantly, and sometimes lack coherence. This is in part because relevant interventions engage numerous legal domains, not least advertising law, criminal law, consumer law, health law, and media regulation. As a result, not only are rules difficult to map, but they are also applied by numerous actors and institutions. Each of these in turn might operate with different assumptions. This makes the implementation of rules challenging to trace, for other regulators and observers alike.

Importantly, tolerance fluctuates over time, which can also generate some inconsistency within jurisdictions. For instance, state concerns over contested health-claims can intensify during public health crises, as we saw during the Covid pandemic, or focus on particular issues as they temporarily gain salience in public discourses ( Atuire *et al.*, 2021). Relevant examples here are extreme diets, contested mental health therapies, or ineffective and dangerous abortion techniques - each involving claims which are more or less problematised over time and in different states. At the same time, and particularly in the current context of political volatility, the relative severity or tolerance shown by state regulators towards contested health claims can also be affected by ideological shifts in governments ( Gagliardone *et al.*, 2021; Lasco & Yu, 2022).

## Health claims, law, and society

Overall, the diversity and fragmented nature of decision-making and its inter-dependency with socio-cultural contexts, makes both the analysis of current practice challenging - and so too the design and implementation of future strategies. Yet, a modest first step is to grasp a more in-depth appreciation of the nature and role of law in this terrain. Our starting point is that this requires a critical approach to understanding the type of social tool that law is.

First, it is paramount to recognise that law incorporates power relations. It draws lines of legitimacy that – like science itself – often embed and perpetuate social inequity, gendered, or racialised prejudice, and colonial relationships ( Monnais, 2021; Seear *et al.*, 2023). In its practice, law is multifaceted, rendered through everyday interactions, and experienced differently across society ( Chua & Engel, 2019). Similarly, both controversial and established health-related claims are produced through social practices that often leverage similar evidentiary techniques ( Prasad, 2021). Indeed, the social debates they trigger frequently illustrate the contingency of what is even deemed legitimate knowledge in the first place ( Fassin, 2021; Whitt, 2009). Concurrently, rather than pre-existing such debates, law itself is co-constitutive of social relations, epistemic norms, and biomedical ontologies – and as such is inherently political ( Biagioli & Pottage, 2021; Cloatre, 2013; Cloatre & Pickersgill, 2021; Pickersgill (in press)).

Exploring the legal regulation of contested claims and knowledges requires careful and cross-disciplinary appreciation of such dynamics. Significant conceptual tools already exist; for instance, from feminist and postcolonial critiques that have encouraged a more granular evaluation of legal interventions as always part of broader patterns of power and dominance by states and some groups within. However, these tools have largely not been applied to the domain of health-related claims and their regulation, despite their pertinence. In particular, questions of belonging, citizenship, and relations of socio-cultural power are crucial to the negotiations of legal boundaries between claims deemed acceptable and those requiring intervention. This includes issues as diverse as who, in a specific context, is considered to be a medical or clinical expert; the place that faith, culture, and beliefs play in health and wider social practices; the relevant role of states in constraining personal expression; the space states need to carve for alternative beliefs; or the role of the states vis-à-vis markets and industries.

The perceived vulnerability of groups and patients, or the relative power and influence of particular industries or particular communities, are examples of factors that impact where and how regulators see the need and possibility of intervention ( Fineman, 2019). Similarly, the perceived urgency of different acute or longer term health crises, can influence how different matters become considered over time as regulatory matters (cf. Ford *et al.*, 2024). To illustrate these considerations further, responses to the Covid pandemic demonstrated how global responses to controversial claims can acquire urgency and trigger significant legal and regulatory shifts that had not previously been deemed necessary or acceptable, despite the very long history of controversial health claims. The growth of the wellness industry is similarly an example of a fast-changing field where boundaries of tolerance, and the legal definitions of what even constitutes a health-related claim, have been challenged by the emergence of new market trends. In other domains, including those where access to health is under strain, or research and knowledge are lacking - such as mental health, reproductive health or chronic illnesses - regulatory responses need to account for the absence of known and/or accessible solutions for patients ( Piña-Romero, 2023).

In understanding and conceptualising legal processes, and legal meanings, it is also crucial to avoid both universalist temptations, and the replication of past mistakes ( Enright *et al.*, 2017). In particular, we are mindful of the dangers of making assumptions about how the law works in the absence of close contextual engagement, or by extrapolating from the observation of a small range of mostly Global North settings ( Atuire, 2023; Cloatre *et al.*, 2023; Reyes-Galindo, 2021). Contexts and their specificities matter to both the nature and the social significance of law, affecting both the particular place of law in society, and how it is likely to be translated into daily practice ( Van Wichelen, 2022). Legal scholarship continues to suffer from being too heavily grounded in Western notions of and perspectives around law, with conceptual and empirical legal research dominated by stories and experiences from a relatively limited set of places. In charting a terrain as complex and context-specific as the regulation of contested claims about health and healthcare, it is essential to adopt, from the outset, a cross-jurisdictional perspective, and contribute to decentering Western, and especially Anglo-American, perspectives on law.

## Consolidating a research agenda

We propose a research agenda aimed at fostering deeper comparative knowledge of the making of legal (in)tolerance with regards to unproven, disproven, or misleading health-related claims, and of its socio-cultural and political dimensions. Our aim is to gain new understanding of decision-making and its underpinning problematisations and ensuing regulatory strategies within this terrain, including whether some voices are more likely to be contested (or granted higher tolerance) than others, what forms of harm are seen as more disruptive (and to whom), and who states implicitly or explicitly speak for when making such decisions (such as commercial actors and afflicted persons). We see such research as a step towards defining and developing fairer and more sustainable regulatory strategies, that are able to protect individuals and communities from harmful health-claims, while carefully balancing fairly competing interests. Our proposed approach is threefold.

First, we need to understand better how laws and regulations formally define the boundaries of acceptability of unproven, disproven, or misleading health-related claims. Part of this entails identifying the key texts, processes, and institutions involved in drawing the complex boundaries between claims that are tolerate and those deemed intolerable. This requires working across diverse and sometimes disconnected fields, and accounting for both formal and less formal sets of criteria and techniques of intervention. Equally important to understand are the particular logics and rationales of different legal strategies is their contextualisation, both in past and present regulatory, policy, and parliamentary debates, and against their broader socio-cultural backdrop.

Second, understanding law and regulation should never stop with a static evaluation of rules. Particularly in this lively and controversial field, it is also essential to explore how rules are enacted and resisted in practice, and how regulatory boundaries are negotiated in everyday scientific, clinical, and personal life. Such attention to the ‘mundane significance’ ( Pickersgill *et al.*, 2011) of routinely regulating contested claims beyond spectacular cases is key to revealing in granular depth how potential discontents are expressed - not only across different jurisdictions, but also health domains.

Third, we must be prepared to imagine more creative alternatives to current strategies ( Cohen & Morgan, 2023; Cooper, 2020; Enright, 2020). A critical exploration of the debates and tensions surrounding the regulation of contested health-related claims must pay particular attention to the effects of law in facilitating or hindering social and health justice. These are effects that critical legal scholars have long called policymakers to be more reflective about ( Harrington, 2018; Jacob & Kirkland, 2020). Building on such critiques and equipped with better knowledge of current patterns of intervention, resistance, and negotiation, we hope to contribute to enabling scholars, policymakers, and communities to collaboratively develop alternative and inclusive legal and regulatory strategies.

## Disclaimer

The views expressed in this article are those of the author(s). Publication in Wellcome Open Research does not imply endorsement by Wellcome.

## Data Availability

No data are associated with this article.
