# Supplementary material for: States, law, and the regulation of controversial health-related claims: consolidating a research agenda between disciplines and contexts
Source: Wellcome Open Res. 2025 Aug 6;10:414. [Version 1] doi: 10.12688/wellcomeopenres.24597.1 (PMC12449687; doi:10.12688/wellcomeopenres.24597.1)
Supplement: Peer review history

# Reviewer report: Reviewer response for version 1

**DOI:** 10.21956/wellcomeopenres.27106.r130165

**Published:** 2025-09-19

**Authors:** Joanna Kempner

**Affiliations:** 1 Rutgers University, New Brunswick, New Jersey, USA

This open letter tackles a pressing question: how should governments regulate unproven, disproven, or misleading health claims without oversimplifying the problem by treating these claims as a simple matter of true or false. Instead, they quite rightly point out that most health claims shift over time. 

 Given that some so-called health-related “misinformation” might be better understood as “contentious knowledge,” I valued the authors’ warning that regulation can do harm--by reinforcing social hierarchies, silencing cultural beliefs, or driving practices underground. Too often, policy discussions focus narrowly on “cracking down” on misinformation and, in the process, lump together dangerous falsehoods with contested ideas that are still up for debate.

 That said, I think the argument could be pushed a little further in some important ways.

 First, fringe or unproven health practices aren’t always just misguided. Sometimes they’re acts of resistance against health systems that neglect or dismiss people. And resistance can be generative. Practices that start out on the margins occasionally spark innovation—or even become mainstream medicine. If regulation is too rigid, we risk shutting down not just harmful practices, but potentially valuable ones.

 Second, the paper raises important questions about tolerance and state power, but I wanted to see more on who actually gets to draw the line. Regulators, courts, professional bodies, and platform moderators aren’t neutral. Do credentialed experts get more leeway than laypeople when making questionable claims? How do protections like freedom of religion in the U.S. reshape what counts as acceptable? These are hard questions, but naming them explicitly would strengthen the analysis.

 Finally, the authors are right to caution against assuming the Global North is the default, but I think the piece could go further. What would it look like to actually compare legal and cultural contexts that are radically different, and build a research agenda from there? A more concrete methodological suggestion would help move that forward.

 Overall, this is a powerful and generative contribution. It pushes the debate about health misinformation away from simplistic fact-checking and toward deeper questions about how law shapes what counts as health knowledge. My comments are meant as sharpening, not criticism: grounding the argument with some prevalence data, taking power dynamics head-on, and drawing out the connection between resistance and innovation could make this letter an even stronger guide for policymakers and scholars alike.

Does the article adequately reference differing views and opinions?

Yes

Are all factual statements correct, and are statements and arguments made adequately supported by citations?

Yes

Is the Open Letter written in accessible language?

Yes

Where applicable, are recommendations and next steps explained clearly for others to follow?

Yes

Is the rationale for the Open Letter provided in sufficient detail?

Yes

Reviewer Expertise:

Sociology of science and medicine

I confirm that I have read this submission and believe that I have an appropriate level of expertise to confirm that it is of an acceptable scientific standard.

# Reviewer report: Reviewer response for version 1

**DOI:** 10.21956/wellcomeopenres.27106.r130179

**Published:** 2025-09-11

**Authors:** Donato Vese

**Affiliations:** 1 University of Pisa Department of Law, Pisa, Tuscany, Italy

The manuscript, 'States, Law and Regulation of Controversial Health Claims: Consolidation of a Research Programme Across Disciplines and Contexts', addresses the challenging topic of disputed claims regarding their reliability in the medical and health field.

 The authors adopt an original perspective by placing themselves outside the true/false dichotomy that is prevalent in specialist scientific literature in this field. In light of these observations, the present study puts forward a research agenda that aims to address this lacuna and serve as a nexus between diverse academic domains.

 A pivotal issue and a foundational concept is tolerance, a subject that the authors examine critically, highlighting the significant variations in state legislation and regulations concerning the definition of what constitutes misleading news and what does not. The authors contend that this discrepancy is particularly evident in terms of inequality in the legal and regulatory approach to be adopted and implemented.

 The conclusions coherently and logically outline the objectives of the proposed agenda, especially for legal scholars.

 My suggestion is to consider integrating Cass Sunstein's studies on freedom of speech

 (e.g. *Liars: Falsehoods and Free Speech in an Age of Deception* by Cass R. Sunstein, Oxford: Oxford University Press, 2021, pp. 192 and more recently: Id., Campus Free Speech

 A Pocket Guide, 2024) even if you believe in a critical sense.

Does the article adequately reference differing views and opinions?

Yes

Are all factual statements correct, and are statements and arguments made adequately supported by citations?

Yes

Is the Open Letter written in accessible language?

Yes

Where applicable, are recommendations and next steps explained clearly for others to follow?

Yes

Is the rationale for the Open Letter provided in sufficient detail?

Yes

Reviewer Expertise:

Administrative Law, Public and Constitutional Law, Law and Economics, European Banking Law, Health Law.

I confirm that I have read this submission and believe that I have an appropriate level of expertise to confirm that it is of an acceptable scientific standard.

## References

[1] Liars: Falsehoods and Free Speech in an Age of Deception by Cass R. Sunstein, Oxford: Oxford University Press, 2021, pp. 192 and more recently: Id., Campus Free Speech A Pocket Guide, 2024.
